# Three-Dimensional Printing of an Apigenin-Loaded Mucoadhesive Film for Tailored Therapy to Oral Leukoplakia and the Chemopreventive Effect on a Rat Model of Oral Carcinogenesis

**DOI:** 10.3390/pharmaceutics14081575

**Published:** 2022-07-28

**Authors:** Hiroyuki Takashima, Tatsuaki Tagami, Shinichiro Kato, Heeju Pae, Tetsuya Ozeki, Yasuyuki Shibuya

**Affiliations:** 1 Department of Oral and Maxillofacial Surgery, Graduate School of Medical Sciences, Nagoya City University, 1, Kawasumi, Mizuho-ku, Nagoya 467-0001, Japan; hiroyuki.tksm.617@gmail.com (H.T.); shintomoyuhi@gmail.com (S.K.); 2 Drug Delivery and Nano Pharmaceutics, Graduate School of Pharmaceutical Sciences, Nagoya City University, 3-1, Tanabe-dori, Mizuho-ku, Nagoya 467-8603, Japan; tagami@phar.nagoya-cu.ac.jp (T.T.); c182045@ed.nagoya-cu.ac.jp (H.P.); ozekit@phar.nagoya-cu.ac.jp (T.O.)

**Keywords:** 3D printing, tailored medicine, oral film, apigenin, semi-solid extrusion, oral potentially malignant disorders (OPMDs), oral leukoplakia

## Abstract

Oral leukoplakia, which presents as white lesions in the oral cavity, including on the tongue, is precancerous in nature. Conservative treatment is preferable, since surgical removal can markedly reduce the patient’s quality of life. In the present study, we focused on the flavonoid apigenin as a potential compound for preventing carcinogenesis, and an apigenin-loaded mucoadhesive oral film was prepared using a three-dimensional (3D) bioprinter (semi-solid extrusion-type 3D printer). Apigenin-loaded printer inks are composed of pharmaceutical excipients (HPMC, CARBOPOL, and Poloxamer), water, and ethanol to dissolve apigenin, and the appropriate viscosity of printer ink after adjusting the ratios allowed for the successful 3D printing of the film. After drying the 3D-printed object, the resulting film was characterized. The chemopreventive effect of the apigenin-loaded film was evaluated using an experimental rat model that had been exposed to 4-nitroquinoline 1-oxide (4NQO) to induce oral carcinogenesis. Treatment with the apigenin-loaded film showed a remarkable chemopreventive effect based on an analysis of the specimen by immunohistostaining. These results suggest that the apigenin-loaded mucoadhesive film may help prevent carcinogenesis. This successful preparation of apigenin-loaded films by a 3D printer provides useful information for automatically fabricating other tailored films (with individual doses and shapes) for patients with oral leukoplakia in a future clinical setting.

## 1. Introduction

Three-dimensional (3D) printing technology has been implemented in many industries, and its application is expanding in various fields, including the medical field [1,2,3]. The process of 3D printing is used for manufacturing explicitly designed objects by successively laminating multiple layers of material. A number of materials, such as plastic, ceramics, metals, and resins, are available for 3D printing and have been utilized for various purposes. The type of 3D printer used depends on the materials involved, which can be in a liquid, semi-solid, or solid state.

One of the advantages of 3D printing is that the object can be designed in advance using a 3D computer-aided design (CAD), which allows for the on-demand manufacture of objects tailored to a specific individual [4]. In pharmaceutics, 3D printing can produce medicine not only of different dosages to meet the clinical needs of each patient but also different complex shapes and structures in order to control the drug release rate and timing, which is not feasible with the conventional mass-manufacturing processes. Such customization has shown potential benefits in patient compliance. Recent reports have described various dosage forms delivered via the oral or parenteral route [5], including tailored polypill tablets [6], floating tablets [7], capsules [8], soft dosage forms [9], orally disintegrating tablets & film [10,11], mucoadhesive film [12,13], suppository formulations [14,15], vaginal rings [16], and ophthalmologic patches [17].

The application of 3D-printing technology holds promise for individualized dental therapy in the oral cavity, which may meet unmet medical needs [18]. Since lesions derived from inflammation and cancer in the oral cavity are diverse among patients, the preparation of 3D-printed objects for surgery, implantation or prostheses is an effective therapeutic approach. Many researchers have proposed new and exciting treatment approaches involving the manufacture of dental restorations in oral and maxillofacial surgery, such as dental implants, temporomandibular joint prostheses, facial epitheses, and crown coping [19,20,21,22,23,24]. Versions of these objects which are 3D-printed can be accurately prepared using a 3D design mediated by imaging techniques, such as magnetic resonance imaging or computed tomography. However, the development of drug formulations in the oral cavity, including the tongue, is still in its infancy, as tinidazole-loaded dental filler [25] and mucoadhesive oral film [13] are reported previously. We believe that the 3D printing of medicine with different dosages and shapes will prove useful for treating individual patients via tailored medicine, as this field has room for growth.

Oral potentially malignant disorders (OPMDs) are the most widely recognized oral lesions with a precancerous nature [26]. The World Health Organization lists 12 lesions as OPMDs [27], and patients with OPMDs have a relatively high risk of malignancy (estimated 5–100 times greater than in the general population [28]). Oral leukoplakia, which presents as white lesions of the oral mucosa in the oral cavity, including the tongue, is a well-recognized OPMD. Long-term outcome of non-surgical treatment in patients with oral leukoplakia is reported [29], and the cumulative 5-year cancer rate with oral leukoplakia is 1.2–14.5%, and the 10-year rate is 2.4–29.0% [30]. While the first choice for the radical cure of this lesion is surgery, the complication of oral carcinoma resection from the oral cavity including the tongue has often resulted in difficulty with eating, the treatment of which involves gastrostomy, thus resulting in a marked reduction in the quality of life. Since no uniform management modality for preventing and treating oral leukoplakia has yet been established [31,32], evidence concerning drug treatment is limited at present, so new compounds to not only treat oral leukoplakia but also prevent oral carcinoma are being explored.

**Figure 1:**
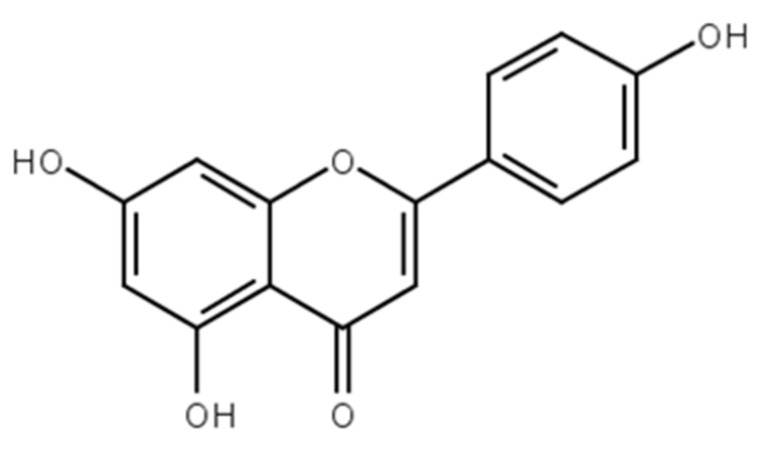
Chemical structure of apigenin (molecular weight: 270.24).

In the current study, we focused on the food-derived flavonoid apigenin (chemical structure, Figure 1), which may be able to prevent oral carcinoma. Flavonoids have multiple pharmacological effects, such as antioxidant activity, anti-inflammation effects, and anticancer effect to tongue squamous cell carcinoma [33]. In our previous study, after screening 21 compounds with a flavone skeleton, we found that apigenin and another compound, luteolin, were able to modulate messenger RNA (mRNA) splicing at the genome-wide level and reduce the viability of several cancer cell lines [34]. mRNA processing (5′ capping, splicing, and 3′ end processing) is an essential step in sustaining various cell functions, such as proliferation, survival, and differentiation [35,36,37], and causing various pathological conditions, such as the onset and progression of carcinoma [38,39]. The potency of apigenin itself may not be strong, but we expected that it would be able to prevent carcinogenesis by acting for an extended period of time.

For these reasons, the apigenin-loaded film was fabricated using a semi-solid material extrusion-type 3D printer, which can extrude viscous materials, including hydrogel and paste. We characterized the 3D-printed oral film containing apigenin and evaluated the chemoprevention potential of the apigenin-loaded film using an experimental precancerous rat model with tongue oral carcinoma induced by 4-nitroquinoline 1-oxide (4NQO).

## 2. Materials and Methods

### 2.1. Materials

Apigenin (95% ≤) and ethanol (99.5%) were purchased from FUJIFILM Wako Pure Chemical Co. (Osaka, Japan). Hydroxypropyl methylcellulose (HPMC; METOLOSE^®^, viscosity of 2% (wt) aqueous solution at 20 °C; 100,000 mPa·s) was supplied by Shin-Etsu Chemical Co. (Tokyo, Japan). Carboxyvinyl polymer (CARBOPOL^®^ 974P) was procured from Lubrizol Co. (Wickliffe, OH, USA). Triblock copolymer consisting of a central hydrophobic block of polypropylene glycol flanked by two hydrophilic blocks of polyethylene glycol (Poloxamer^®^ 407) was provided by BASF Co. (Land Rheinland-Pfalz, Germany). 4NQO was purchased from Tokyo Chemical Industry Co., Ltd. (Tokyo, Japan).

### 2.2. Preparation of Inks for Apigenin-Loaded Film

Printer inks were prepared via an approach modified from our previous report [13]. Five types of inks were prepared with different ratios of water to ethanol (Table 1). Firstly, ethanol and a stirring bar were added to a screw-top glass vial and placed in a water bath at 65 °C. Appropriate amounts of apigenin powder (2.5 mg) were then added to the vial while stirring. After dissolution, water, CARBOPOL^®^ (200 mg), Poloxamer^®^ (200 mg), and HPMC (200 mg) were added to the vial in turn while stirring. The vial was held at room temperature overnight.

### 2.3. Viscous Property of Printer Ink

The viscosity and sheer stress of each sample was measured by a cone-plate viscometer (Brookfield viscometer, DV2T; Brookfield, MA, USA) using a CPE-40 spindle at 25 °C. The rotation speed was set to gradually increase every 30 s (0.1, 0.2, 0.5, 1, 2, 5, 10, 20, 50, 100, and 200 rpm).

### 2.4. 3D Design and Fabrication of Apigenin-Loaded Film

The 3D design of the film was designed using 123D Design, a 3D CAD software program (Autodesk Inc., San Rafael, CA, USA). The size was set to 20 mm (length) × 15 mm (width) × 1 mm (height). The 3D-printing conditions were set using the *Slic3r* slicer software program (GNU General Public License). The printer ink, prepared as described in the previous section, was carefully loaded into a syringe with a 27G nozzle and then set in the 3D bioprinter (INKREDIBLE; CELLINK, Gothenburg, Sweden). A clear polypropylene sheet was fixed on the 3D bioprinter stage to support the 3D printing, which was achieved by the extrusion of printer ink from the nozzle. The extrusion of the ink was controlled using air pressure through a pump. The pressure was set to 90 kPa. The printed hydrogel was air-dried at room temperature overnight.

### 2.5. Measurement of Film Weights and Thickness

The weights of film samples were measured on an electric balance (*n* = 5). The thickness of film samples was measured to the nearest 0.001 mm using an MDC-25MX (Mitutoyo Co., Kanagawa, Japan). We conducted the measurements at three randomly selected points (*n* = 5).

### 2.6. Differential Scanning Calorimetry (DSC)

The DSC peaks of the film, apigenin, and excipient were measured by a DSC-60 differential scanning calorimeter (SHIMADZU Co., Kyoto, Japan). About 2 mg of samples were placed in the bottom of the sample pan, and, then, the temperature was ramped up from 30 to 400 °C at a rate of 10 °C/min. In all DSC experiments, nitrogen gas was used as the purge gas at a flow rate of 20 mL/min.

### 2.7. Powder X-ray Diffraction (XRD)

Powder XRD patterns of film and powder samples were analyzed. They were obtained using a MiniFlex 600 (Rigaku Co., Tokyo, Japan) by irradiating with Cu-*Kα* X-rays. The tube voltage and amperage were 40 kV and 15 mA, respectively. Samples were scanned from a 2θ of 3° to 40°.

### 2.8. Dissolution Test

Artificial saliva (8 mL) was added into the screw-top glass vial, and the temperature of the solution was maintained at 37 °C using a water bath incubator shaker (SN-60SD; Nissinrika, Tokyo, Japan). Artificial saliva was prepared using the same contents as in the commercially available artificial saliva, Salivate^®^ Aerosol (TEIJIN PHARMA, Tokyo, Japan). The apigenin-loaded film was added, and the screwed vial was shaken horizontally with the speed scale set to 6 (corresponding to 150 rpm). At appropriate points of time, 500 µL of sample solution was collected, and the same volume of artificial saliva was replenished. Ethanol was added to the collected aliquots to bring the solution to 50% ethanol before the measurement. The concentration of apigenin solution was determined by measuring the absorbance of each aliquot at 266 nm using a UV–visible spectrometer (UV-1800; SHIMADZU Co., Kyoto, Japan). A series of apigenin standard solutions dissolved in 50% ethanol (1, 3, 5, 10, 20, 30, and 50 ppm) were prepared and measured using a UV-1800 spectrophotometer (SHIMADZU Co.).

### 2.9. In Vivo Chemoprevention Potential of the Apigenin-Loaded Film

#### 2.9.1. Animal Experimental Protocol

6-week-old Sprague Dawley (SD) rats, were obtained from Japan SLC, Inc. (Shizuoka, Japan). This series of animal experiments was approved by the Nagoya City University Graduate School of Medicine Animal Ethics Committee.

As in previous reports [40,41,42], we induced the formation of oral lesions on the tongues of rats after the administration of 4NQO solution (20 ppm) every day for 8 weeks. The animals were separated into 2 groups as follows: Group 1 (CTRL) rats received 4NQO solution for 8 weeks and no treatment (*n* = 6); Group 2 (Film) rats received 4NQO treatment for 8 weeks and then had the apigenin-loaded film applied to the tongue twice per week (*n* = 6). The film was further divided into four sections and applied to the rats’ tongue after anesthesia with an anesthetic mixture of medetomidine, midazolam, and butorphanol. An hour later, we relieved the anesthesia by injecting antisedan. All rats were sacrificed at 22 weeks after the apigenin-loaded film application. The rats that died during the process were evaluated immediately using the tongue specimen extracted at death.

#### 2.9.2. Histopathological Examination

After the rats were sacrificed, their tongues were fixed in 10% buffered formalin. All tongue specimens were embedded in paraffin blocks and stained with hematoxylin and eosin (H&E) by the Tokai Cytopathology Institute (Gifu, Japan). Histopathological evaluations in this study were performed by light microscopy. The tongue sections were graded as normal, hyperplasia, dysplasia, and carcinoma per animal according to El-Rouby et al. and Ribeiro et al. [43,44].

#### 2.9.3. Immunohistochemistry (IHC)

IHC assessments were performed in the present study to determine the effect of apigenin on the expression of tumor and inflammation markers—Ki-67(MIB-1), nuclear factor kappa B (NF-κB), and 8-hydroxy-2-deoxyguanosine (8-OHdG)—in the tongue tissue sections of the SD rats. As with H&E staining, IHC was also performed by the Tokai Cytopathology Institute. IHC expression analyses were performed to assess the localization and to compare the percentages of positively stained areas between Group 1 (CTRL) and Group 2 (Film).

The immunostained sections were digitalized using a Nanozoomer S60 (Hamamatsu Photonics, Shizuoka, Japan). Five fields at ×400 magnification, including hot spots, were selected from each sample. The color density and white balance were standardized. The results were assessed using the Image J software program (National Institutes of Health, Bethesda, MD, USA) with an IHC toolbox plugin, based on the report by Al-Afifi et al. [41]. In brief, the semi-automatic color selection and automatic statistical color detection model functions prebuilt into Image J’s IHC Toolbox allow for the evaluation of the percentage of positive (brown) staining. A threshold was set, at which staining could be quantified with positive staining. This was duplicated in all images for a comparison, and all data were analyzed by the SPSS software program, version 26 (SPSS Inc., Chicago, IL, USA). *p* < 0.05 was considered to be significant.

## 3. Results and Discussion

The technology of 3D printing has been expected to be useful for generating personalized medications due to its unique manufacturing ability and high flexibility. Since a semi-solid extrusion-type 3D printer utilizes hydrogel and paste, a 3D bioprinter that uses cell-based hydrogel has been used for the production of tissue containing various cells in the field of regeneration therapy, and some 3D printers have been targeted for use in clinical trials [45,46]. As 3D printers can prepare the desired object automatically, we feel that the application of 3D-printing technology for the preparation of small batches of certain dosage forms, including our film formulation, may be feasible in the future.

In the present study, we attempted to fabricate a mucoadhesive film containing apigenin, a dosage form that can be applied to sites with precancerous tissue on the tongue, such as leukoplakia. The film formulation comprised several pharmaceutical excipients (HPMC, Carbopol, and Poloxamer) as shown in Table 1. HPMC was selected as the typical thickener to mainly generate viscosity. Indeed, an HPMC-based hydrogel was used to prepare a 3D-printed film formulation previously [13]. The viscosity was clearly dependent on the HPMC concentration, and the shear-thinning property was deemed suitable for extrusion from the nozzle of a 3D printer. Based on a previous report, we considered the possibility of utilizing 3D printing for actual intraoral lesions. Carbopol was then further incorporated into the printer ink in this study. Carbopol offers a better mucoadhesive force than other polymers [47], and we anticipated that this excipient would play an essential role in increasing the mucoadhesive properties and promoting retention on the tongue during animal experiments. Poloxamer, a relatively safe hydrophilic surfactant, has been widely used to promote the solubilization of poorly water-soluble drugs. We anticipated the solubilization of apigenin during the process of drug dissolution. While these combinations of pharmaceutical excipients have been used previously to produce mucoadhesive tablets and oral films, their properties with regard to 3D printing have been investigated by few groups [48,49]. For this reason, we started off with investigating the properties of the printer ink, as described below.

### 3.1. Rheological Property of Printer Ink

**Figure 2:**
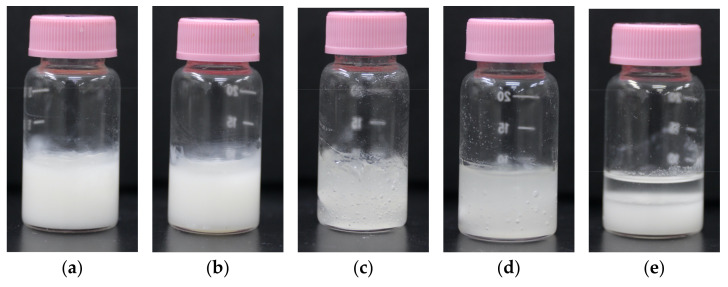
Photos of printer inks: (**a**) Formulation A, (**b**) Formulation B, (**c**) Formulation C, (**d**) Formulation D, (**e**) Formulation E.

**Figure 3:**
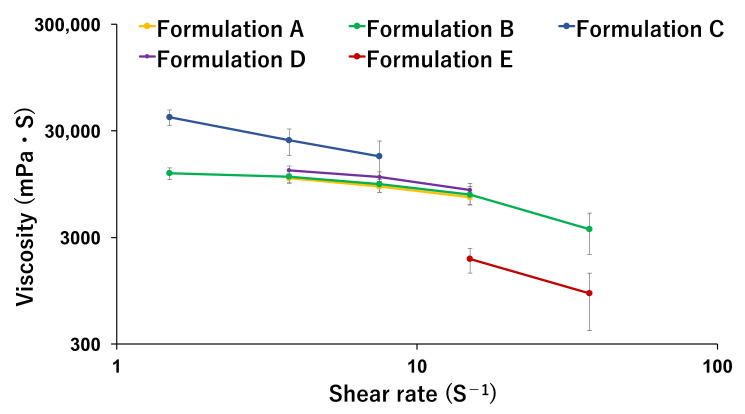
The viscosity of printer ink with different water/ethanol ratios. The composition is described in Table 1. The data are shown as the means ± SD (*n* = 3).

Five formulations with different ratios of water and ethanol were prepared and compared as shown in Table 1. Apigenin is a poorly water-soluble compound, so the incorporation of ethanol into printer ink was expected to be effective in dissolving it. Furthermore, the incorporation of ethanol has other advantages as well, as the formulation can accelerate the drying process after 3D printing. The appearance and viscosity of the resulting inks are shown in Figure 2 and Figure 3. The viscosity decreased in the following order: Formulation C > Formulation D > Formulation A and B > Formulation E. Other reports mentioned an HPMC-based hydro-alcoholic gel for a semi-solid extrusion-type 3D printer. Li et al. prepared an HPMC-based hydro-alcoholic gel (27% water/73% ethanol [*v/v*]) to dissolve dipyridamole, which they further mixed with a powder sample to produce a paste sample for tablets [7]. Zheng et al. also prepared tablets using an HPMC-based hydro-alcoholic gel [50], mentioning that the viscosity was decreased by increasing the proportion of ethanol. In contrast, Formulation C exhibited the highest viscosity in our study (Figure 3). We guessed that excess ethanol may have limited the dissolution of HPMC into water, and the formation of an HPMC-based hydrogel was not adequate. Formulation E, which contained ethanol only, showed phase separation. Since HPMC and other pharmaceutical excipients eventually precipitate and the property of HPMC as a thickener is lost, Formulation E showed the lowest viscosity. Notably, Formulations A and B were cloudier than Formulations C and D (Figure 2). This is due to the presence of suspension of apigenin. Thereafter, we found some bubbles in the printer ink. Bubbles in the printer ink can affect the accuracy of measuring the viscosity and may also be associated with problems in the 3D printing. In this case, we carefully collected the printer ink manually so that the syringe did not include any large bubbles. Gently centrifuging the syringe (e.g., 500 g, 5 min) is an alternative method for efficiently removing any bubbles [17].

Based on the above findings concerning viscosity, Formulation C was deemed to have the most appropriate viscosity and shear-thinning property and, thus, was used for 3D printing. The printer ink flowed well with the pressure during extrusion while avoiding collapsing once laid down in its intended shape. Additionally, as the ink with a high ethanol content (Formulation D and E) may cause nozzle-tip-drying issues (ink clogging), we decided to use Formulation C.

### 3.2. Characterization of Apigenin-Loaded Film

#### 3.2.1. General Characteristics of Apigenin-Loaded Film

**Figure 4:**
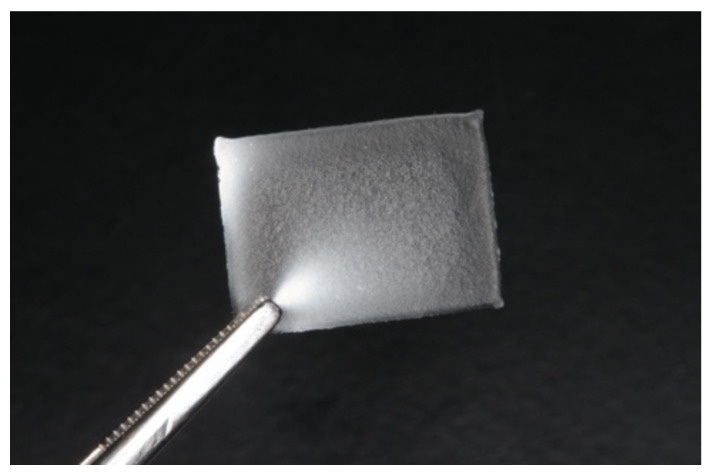
Photo of the apigenin-loaded film. The preparation method was described in detail in the Section 2.

As shown in Figure 4, the apigenin-loaded clear film was obtained successfully. The film had a slightly yellowish color due to the innate color of apigenin. The deviation in the weight and thickness was less than 10% (Table 2), indicating that the ink formulation seemed suitable for 3D printing, and the inclusion of ethanol in the printer ink helped accelerate the drying process. The film had an elastic property, and when it was bent, it returned to its original shape easily.

We speculate that this elastic film may be suitable for application to OPMDs, which occur in various locations in the oral cavity, such as the buccal mucosa, the tongue, the lip, and the floor of the mouth [51]. Mechanical irritation is a risk factor in the development of leukoplakia and malignancy [29]. The oral cavity moves unconsciously at various times, such as during salivary swallowing which dysfunction is a key concern of oral health in old adult [52]. Although future studies regarding the assessment of mechanical properties are necessary, the film was stretchable to some extent and could tolerate such movements. However, the apigenin-loaded film is considered to have an adhesive property and softening property due to HPMC and Carbopol, respectively [47], when it absorbed moisture such as saliva. These properties made it difficult to be removed by slight movements, so the effect of the medicinal agent was expected to last for a relatively long period with minimum mechanical strength. We consider that the influence of the slightly rough surface of this film on mechanical irritation is minimal compared to typical mechanical strength such as physical contact of the tongue with an ill-fitting denture and/or teeth with sharp edges. These properties of the film contributed to the reduction in mechanical stress overall.

#### 3.2.2. DSC

**Figure 5:**
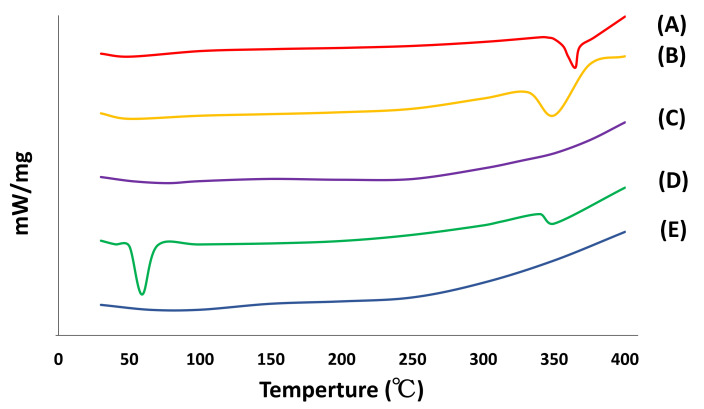
DSC curves of apigenin, pharmaceutical excipients, and the apigenin-loaded film. (A), Apigenin; (B), HPMC; (C), Carbopol; (D), Poloxamer; (E), apigenin-loaded film.

To determine the potential changes in the crystalline state of apigenin, excipients, and the apigenin-loaded film, we examined the DSC curves. The results are shown in Figure 5. Apigenin bulk powder had a single sharp endothermic peak at 365.01 °C corresponding to the melting point of apigenin and indicating its crystalline nature. HPMC and Carbopol had an endothermic peak around 350 °C. Poloxamer also showed a single sharp peak at 59.0 °C as its melting point. These findings were similar to values in previous reports [53,54,55,56]. According to the DSC findings of the apigenin-loaded film, no major thermal events were observed. These results suggested that the optimized film formulation might be in an amorphous state [57]. It is likely that the drug is present in the amorphous phase, which facilitates dissolution, and may be homogeneously dispersed in the film, although the amount of apigenin is less than its detection limit.

#### 3.2.3. XRD

**Figure 6:**
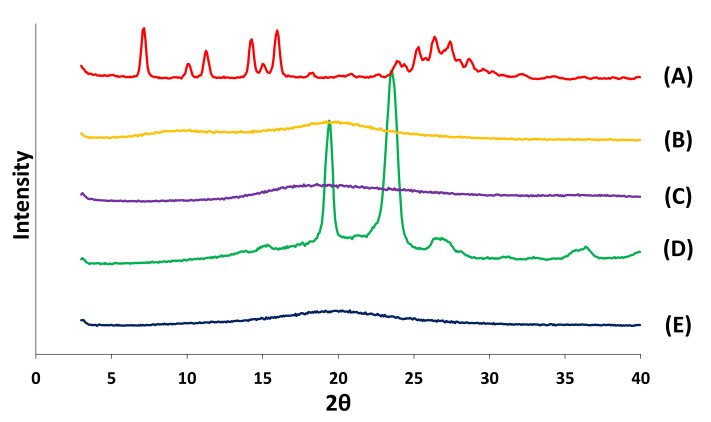
XRD patterns of apigenin, pharmaceutical excipients, and the apigenin-loaded film. (A), Apigenin; (B), HPMC; (C), Carbopol; (D), Poloxamer; (E), apigenin-loaded film.

The XRD diffractograms of apigenin, HPMC, Carbopol, Poloxamer, and the apigenin-loaded film are shown in Figure 6. XRD confirmed the results of the DSC analysis. Distinct peaks of apigenin in the diffractograms were indicated at diffraction 2θ values as follows: 7.16°, 10.12°, 11.24°, 14.28°, 15.08°, 15.96°, 18.12°, 23.88°, 26.36°, 27.40°, and 28.68°, indicating that apigenin was present in a crystalline form. Poloxamer displayed sharp peaks at 2θ values of 19.40° and 23.48°. These results were similar to those of previous reports [53,54,55,56]. HPMC and Carbopol did not have any sharp peaks, and the XRD pattern of the apigenin-loaded film did not show any characteristic peaks either, suggesting that the structure of the apigenin-loaded film was amorphous.

#### 3.2.4. Dissolution Test

**Figure 7:**
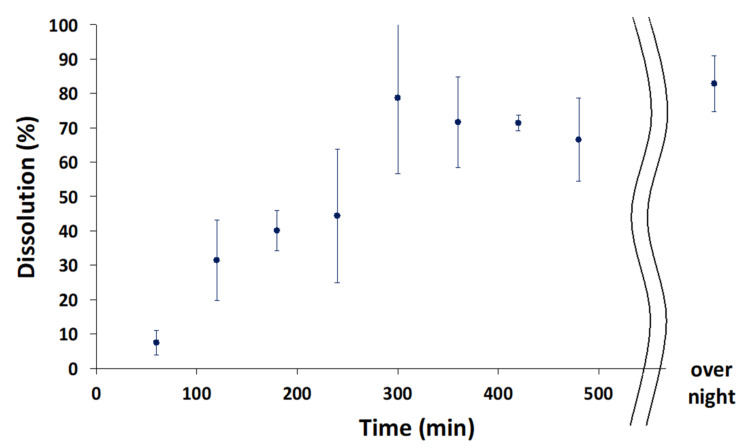
Drug dissolution profiles. The data show the means ± SD (*n* = 3).

An in vitro drug dissolution test was conducted as shown in Figure 7. We used the same components as those in artificial saliva for the drug dissolution test, and the glass vials containing samples were shaken at 37 °C to partially imitate the environment of the oral cavity with movement. The apigenin-loaded film started to gradually release the drug, and nearly 80% had been released from the film within 300 min, eventually eluting 82.9% ± 8.1% of the total apigenin. The HPMC-based film became swollen by absorbing saliva from the oral cavity and released the drug locally in a controlled manner. Although further studies will be necessary, the amount of HPMC can be altered to control the drug release, since the drug release rate was dependent on the amount of HPMC in the film [13].

The drug dissolution profile, unexpectedly, had high deviation. One of the possible reasons for this may be due to the heterogeneity of apigenin in the film, which resulted in a drug release profile with a high deviation. The second possible reason is the experimental conditions associated with shaking the glass vial. We used a relatively small volume of artificial saliva (8 mL) to partially imitate the oral cavity and, then, the film was folded during the subsequent vigorous shaking which may have affected the drug release.

As the apigenin-loaded film can exert a biological effect slowly over a long period of time by topical application to the oral cavity, it may be suitable for application before bedtime. Indeed, the adhesive property of the apigenin-loaded film will likely be able to avoid being shifted around by jaw clenching or sleep-talking in patients. In addition, since the film is a food compound, it is expected to be harmless, even if patients were to accidentally swallow the film.

### 3.3. Chemoprevention for the Rat Tongue Carcinoma Induced by 4NQO

Animal models are useful for determining prophylactic efficacy before embarking on a clinical trial. In the present study, a 4NQO-induced rat oral carcinogenesis model was adopted for the assessment of the chemopreventive effect of apigenin. 4NQO is a water-soluble quinolone derivative found in cigarette smoke [40,58]. Rat oral lesions induced by 4NQO are comparable to those seen in humans, as 4NQO induces sequential stages of carcinogenesis (hyperplasia, dysplasia, and endophytic or exophytic tongue tumors) [58,59].

#### 3.3.1. General Observation

**Figure 8:**
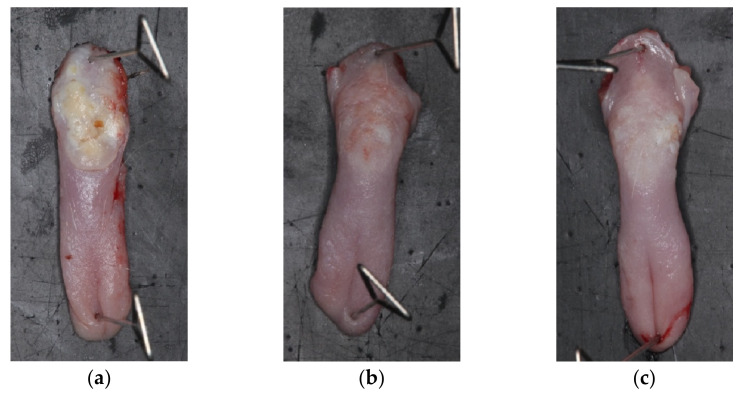
Excised tongue specimens from each group: (**a**) CTRL group; (**b**) Film group; (**c**) Film group (tumor at the left lateral border of the tongue).

CTRL (untreated) and the apigenin-loaded films were compared in this study. The typical appearance of each group after the treatment is shown in Figure 8. The CTRL group showed large tumors in the dorsal region of the posterior tongue. Previous reports have described similar tumors occurring in the dorsal region of the posterior tongue [40,41,59]. The group treated with the apigenin-loaded film, conversely, did not develop large tumors.

The influence of treatment on the food intake and body weight was also investigated. Mean weekly food intakes after the apigenin-loaded film initiation (139.4 ± 12.0 g/rat in the CTRL group, 150.2 ± 10.9 g/rat in the Film group) were not significantly different between groups. The survival rate and final body weight are shown in Table 3. Weight loss may be a useful indicator of the clinical progression of carcinoma [60]. The 4NQO-induced rat oral carcinogenesis model may show substantial body weight loss due to the development of oral carcinoma, a lack of appetite, an inability to eat, and an increase in the metabolic rate [61]. In the present experiment, a similar decrease in body weight was observed in the CTRL group, especially around the end of the experiment (data not shown), and the final body weight of the CTRL group was lower than that of the group treated with the apigenin-loaded film. Due to the progression of carcinoma, one rat in the CTRL group was found dead prior to the termination of the study, whereas all rats in the group treated with the apigenin-loaded film survived to the endpoint.

#### 3.3.2. Histopathological Assessment

**Figure 9:**
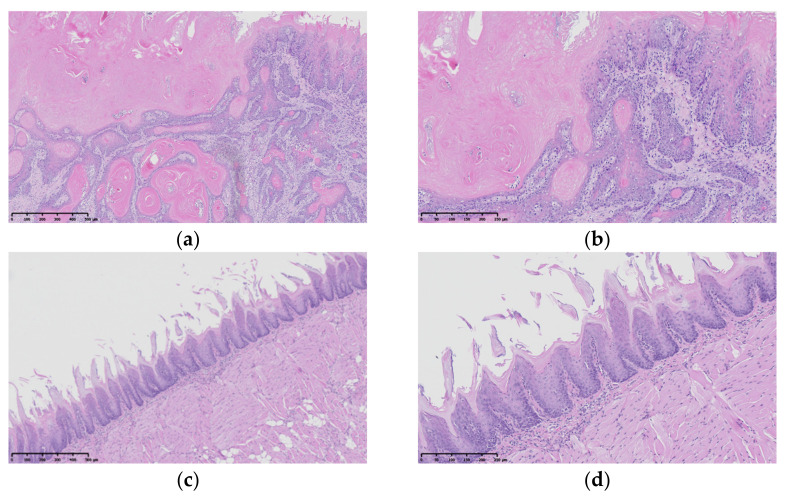
Histopathological assessments of tongues following H&E staining: (**a**) CTRL group (Mic. Mag.; ×100); (**b**) CTRL group (Mic. Mag.; ×200); (**c**) Film group (Mic. Mag.; ×100); (**d**) Film group (Mic. Mag.; ×200).

The carcinoma incidence in the rats’ tongues at 22 weeks is reported to be extremely reproducible (85.7%) for the rats administered 4NQO solution for 8 weeks at 20 ppm [59]. A significant reduction in the tumor incidence on the dorsal tongue was observed in the apigenin-loaded-film-treated group (50% tumor incidence vs. 100% in the CTRL group) (Table 4, Figure 9). In the film-treated group, small tumors developed at the root and lateral border of the tongue due to insufficient coverage of the film (Figure 8c). We, therefore, believe that the apigenin-loaded film has an inhibitory effect on tumor development, and this effect may be enhanced if the frequency of treatment could be improved (e.g., increased to daily) compared to the current experimental condition (twice per week). Under the current conditions of this animal experiment, the effect was limited, as rats might have eaten or removed the film once awakened. The actual application of this treatment to human patients may result in better therapeutic efficacy due to the retention of the film for a longer period of time.

#### 3.3.3. IHC Assessment

**Figure 10:**
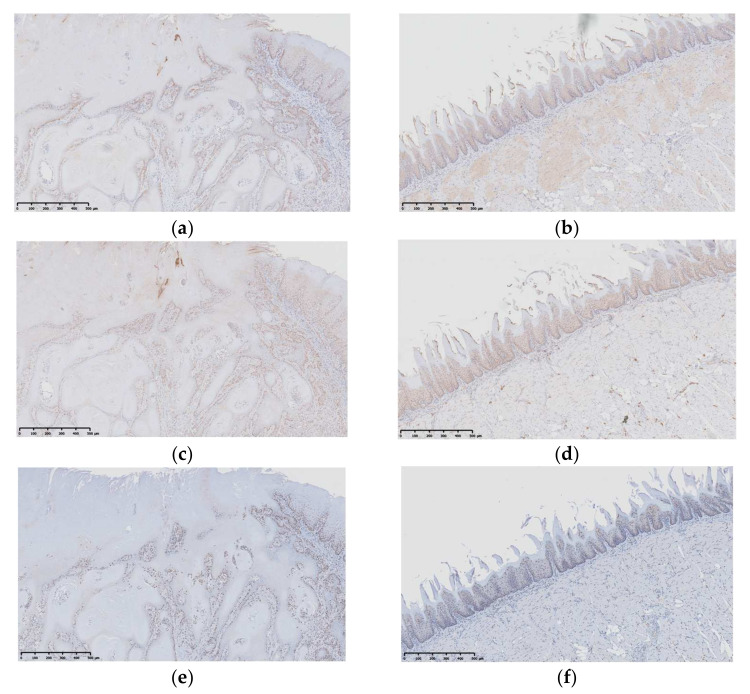
Photomicrographs of IHC reactions (Mic. Mag.; ×200): (**a**) Ki-67, CTRL group; (**b**) Ki-67, Film group; (**c**) NF-κB, CTRL group; (**d**) NF-κB, Film group; (**e**) 8-OHdG, CTRL group; (**f**) 8-OHdG, Film group.

IHC of the selected stains was performed to evaluate and compare the percentage of positively stained areas for Ki-67, NF-κB, and 8-OHdG between the CTRL group and Film group (Figure 10 and Table 5). Apigenin is expected to prevent the development of oral carcinoma by regulating the cellular-function processes and homeostasis, such as via mRNA splicing [34]; in addition, the compound can arrest the cell cycle of oral squamous-cell carcinoma [62]. Ki-67 is widely used as a reliable marker for clarifying the cell proliferation in neoplastic tissues [63,64]. In the present study, the percentage area of Ki-67 immunoexpression in the film-treated group was significantly lower than that in the CTRL group (Figure 10a,b, Table 5). The decrease in Ki-67 is presumably due to the carcinogenic inhibition effect by the apigenin-loaded film. It has been suggested that Ki-67 has improved our understanding of the biological behavior and prognosis of carcinoma, and it reportedly correlates directly with carcinoma with malignant transformation [65]. Ki-67 immunoexpression was detected in the basal layer of tongue cells in the normal tongue. Then the expression of Ki-67 was higher in human tissue section of poorly differentiated oral squamous cell carcinoma [66]. These previous findings support our current results.

The transcription factor NF-κB is an early response gene that promotes the expression of inflammatory cytokines and other factors [67]. It plays an important role in carcinogenesis, and aberrant regulation of NF-κB has been observed in many cancers [68]. There was no significant difference in the NF-κB levels between the CTRL group and the Film group (Figure 10c,d, Table 5), although the Film group tended to have a lower expression. The current experiment using 4NQO was conducted for a relatively long period of time, allowing for ample inflammation of the tongue tissue to occur in both groups, thereby resulting in the expression of NF-κB.

An oxidative product of DNA damage, 8-OHdG, is induced by reactive oxygen species and often used to evaluate oxidative stress, which can lead to cell damage, induced by an imbalance between the antioxidant and prooxidant systems [69]. Various diseases, including OPMDs—such as lichen planus, leukoplakia, pemphigus vulgaris, periodontitis, and oral cancer—have been linked to cellular damage associated with increased oxidative stress [70,71,72,73]. In the present study, the percentages of areas positive for 8-OHdG following treatment with the apigenin-loaded film were significantly lower than in the CTRL group, indicating that apigenin has the ability to reduce reactive oxygen, as a flavonoid. These results suggest that the apigenin molecules were dissolved from the 3D-printed film and prevented the oxidization of tongue tissue induced by 4NQO, thereby exerting a chemopreventive effect.

## 4. Conclusions

In conclusion, the 3D printing of oral films containing apigenin was conducted successfully using optimized printer ink with good viscosity with a semi-solid-type 3D printer. This apigenin-loaded mucoadhesive film was effective in a 4NQO-induced oral carcinogenesis rat model, resulting in the prevention of carcinogenesis. The use of an oral film with elastic and mucoadhesive properties is likely ideal for the treatment of OPMDs, including oral leukoplakia. Although further experiments will be necessary, the application of 3D-printing technology to tailored film formulations may hold promise in the treatment of diseases in the oral cavity. In addition, it may be possible to adjust the shape and drug dosage based on the imaging data of leukoplakia in the clinical setting.

## Figures and Tables

**Table 1:** Drug formulations as printer inks.

Composition	Apigenin (mg)	Ethanol (mL)	Water (mL)	CARBOPOL (mg)	Poloxamer (mg)	HPMC (mg)
Formulation A	2.5	0	9.4	200	200	200
Formulation B	2.5	2.35	7.05	200	200	200
Formulation C	2.5	4.7	4.7	200	200	200
Formulation D	2.5	7.05	2.35	200	200	200
Formulation E	2.5	9.4	0	200	200	200

**Table 2:** The mean weight, thickness, and estimated drug content of the three-dimensionally printed film. The drug content in the film was calculated from the weight and film composition. The data are shown as the mean ± SD (*n* = 5).

Weight (mg)	Thickness (μm)	Drug (μg)
159.7 ± 11.5	39.3 ± 2.7	163.0 ± 11.4

**Table 3:** The survival rate and final body weight of rats after treatment. ^a^ Data are given as the mean ± SD (*n* = 6).

Group	Survival Rate	Final Body Weight (g) ^a^
CTRL	5/6 (83.3%)	484.4 ± 94.2
Apigenin-loaded film	6/6 (100%)	576.6 ± 73.1

**Table 4:** In vivo histopathological assessments of rat tongues. * *p* < 0.05 vs. CTRL (chi-square test).

Group	Normal	Hyperplasia	Dysplasia	Carcinoma
CTRL	0 (0%)	0 (0%)	0 (0%)	6 (100%)
Apigenin-loaded film	0 (0%)	0 (0%)	3 (50%)	3 * (50%)

**Table 5:** The comparison of the percentage of positively stained areas. Data are given as the mean ± SD (*n* = 6). * *p* < 0.05 vs. CTRL (*t*-test).

IHC Maker	Group	Mean ± SD (%)	*p* Value
Ki-67	CTRL	4.42 ± 2.40	<0.0001 *
	Apigenin-loaded film	2.54 ± 1.08	
NF-κB	CTRL	13.77 ± 3.83	0.643
	Apigenin-loaded film	12.30 ± 4.39	
8-OHdG	CTRL	12.20 ± 1.96	0.014 *
	Apigenin-loaded film	8.34 ± 4.05	
